# Evaluation of Spatial Distribution of Pulse Blue Butterfly (*Lampides boeticus*), Pest of Legume Crops, in Response to Climate Change

**DOI:** 10.3390/insects16080826

**Published:** 2025-08-08

**Authors:** Jeong Ho Hwang, Sunhee Yoon, Wang-Hee Lee

**Affiliations:** 1 Wetland Center, National Institute of Ecology, Changnyeong 50303, Republic of Korea; chsh123@nie.re.kr; 2 Department of Smart Agriculture Systems, Chungnam National University, Daejeon 34134, Republic of Korea; yoonsh1604@gmail.com; 3 Department of Smart Agriculture Systems Machinery Engineering, Chungnam National University, Daejeon 34134, Republic of Korea

**Keywords:** Lycaenidae, legume pest, potential distribution, occurrence possibility, species distribution modeling

## Abstract

The potential distribution of the pulse blue butterfly, *Lampides boeticus* (Lepidoptera: Lycaenidae), was determined using MaxEnt, random forest, and ensemble models. The results indicate that most tropical, subtropical, and some temperate regions are suitable habitats. Climate change is projected to expand the species’ habitat northward in the Northern Hemisphere. Predicted distributions aligned well with the known occurrence records for the species. The minimum temperature of the coldest month was the climatic variable that most strongly influenced the distribution of *L. boeticus*. As a tropical and subtropical species, it is assumed that cold temperatures are the main factor limiting its habitat range. Because the potential distribution of this pest covers major pulse cultivation areas under both current and future climate scenarios, these findings highlight the urgent need for developing a sustainable pest management strategy.

## Simple Summary

*Lampides boeticus*, commonly known as the pulse blue butterfly, is a significant pest of legume crops. Its habitat is shifting as a direct consequence of climate change, which necessitates an assessment of its potential future distribution. This study investigated the potential distribution of *L. boeticus* using MaxEnt, random forest, and ensemble models. The predicted distributions aligned well with the species’ recorded occurrences. Results showed that climate change is projected to broaden its habitat, an effect primarily driven by the minimum temperature of the coldest month as shown in variable contribution. As a tropical and subtropical species, it is assumed that cold temperatures are the main factor limiting its habitat range. The models predict that the species’ potential range will overlap with major pulse cultivation areas, regardless of climate change. This finding underscores the urgent need to establish a sustainable pest management strategy for this pest.

## 1. Introduction

The pulse blue butterfly (*Lampides boeticus* Linnaeus, 1767) is distributed across Africa, Eurasia, and Oceania, as well as on islands in the Pacific, Atlantic, and Indian Oceans. Its range spans tropical to temperate zones, covering nearly all continents except the Americas [1]. This butterfly feeds on more than 100 plant species, primarily legumes, and is a rare example within the Lycaenidae family known for its exceptional migratory abilities [1,2,3,4]. Consequently, with few non-climatic habitat limitations, its potential to impact local agriculture is significant.

Research on this butterfly has primarily been conducted in India, where it is a recognized pest of legume crops like cowpeas and field peas. However, damage has also been reported in Iran, Iraq, Egypt, Kenya, Nigeria, Thailand, Vietnam, France, and Japan [5,6,7,8,9,10,11,12,13]. In severe cases, this species can cause over 50% of damage to legume crop production [14,15]. In South Korea, for instance, it was designated a climate change bioindicator species by the Ministry of Environment following its recent establishment in the southern region and subsequent damage to *Lablab purpureus* (L.) [16]. Similarly, in the UK, more frequent sightings are attributed to the northward expansion of its distribution range as a result of climate change [17]. The documented damage to agriculture and habitat shifts driven by climate change necessitate a predictive assessment of this pest’s potential distribution to develop effective countermeasures against its spread [18,19].

Species distribution modeling (SDM) is a common method for assessing potential habitats and their shifts in response to environmental factors. Among various SDM approaches, machine learning-based (or correlative) models are widely used. Their advantages over mechanistic models include their reliance on occurrence-only data, the ability to incorporate diverse environmental layers, and the use of objective evaluation metrics [20]. Although several SDM algorithms exist, MaxEnt is a well-known tool for evaluating potential distributions using only occurrence data [21]. Based on the principle of maximum entropy, the model identifies environmental conditions at known occurrence locations and extrapolates to predict other suitable areas. MaxEnt has demonstrated strong performance even with relatively small occurrence datasets [22,23]. Consequently, recent SDM studies have utilized MaxEnt to evaluate potential risks from crop pests, including those attacking legumes [18,19]. Another powerful tool is the random forest (RF) algorithm, a machine learning classifier known for its high performance and robust identification of potential species distributions in computational ecology [24,25].

*L. boeticus* is a damaging pest of legume crops, and its impact is expected to increase as climate change expands its suitable habitat and potentially its population density. However, to the best of our knowledge, no previous study has evaluated the global distribution and habitat suitability of this species. Therefore, this study aimed to model the occurrence probability and map the potential distribution of *L. boeticus* under current and future climate scenarios to identify areas at risk. These results can serve as crucial data for understanding the threats posed by *L. boeticus* and for developing management strategies to mitigate its impact.

## 2. Materials and Methods

### 2.1. Occurrence and Background Data Processing

Occurrence records for *L. boeticus* were obtained from two publicly available databases. The Centre for Agriculture and Bioscience International (CABI) reports the species’ distribution across Afro-Eurasia, Oceania, and the Pacific Islands [26]. The Global Biodiversity Information Facility (GBIF) provided georeferenced coordinates for model development, with a distribution largely consistent with that reported by CABI [27].

**Figure 1:**
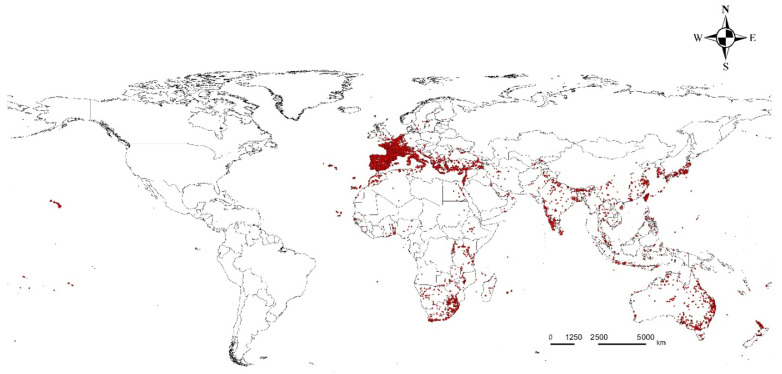
Occurrence records for *L. boeticus*.

A total of 44,341 coordinates were initially obtained from GBIF [27]. Records from the American continents were removed, as they could not be cross validated with data from CABI or a previous study [1,26]. Then, to eliminate redundant records and sampling bias resulting from uneven sampling density, spatial filtering was applied with a 25 km radius buffer, taking into account the climate heterogeneity at the occurrence points. This was accomplished using the SDM toolbox in ArcGIS version 10.4.1 (ESRI). This process resulted in a final dataset of 3669 points for model development (Figure 1) [28,29]. For background data (pseudo-absence points), we generated a biased background file using the kernel density function provided by the R software [30,31,32]. This was performed to weigh occurrence records that could account for uneven sampling density [33].

### 2.2. Model Variables and Their Selection

Assuming that several decades represent an insufficient timeframe for significant evolutionary adaptation, we used historical bioclimatic data to model the current potential distribution of *L. boeticus* [34]. The historical climate data (the average maximum temperature, average minimum temperature, and precipitation) spanned 1992–2021 and elevation data was obtained from WorldClim (www.worldclim.org, accessed on 30 January 2024) [35,36,37]. The historical climate data collected was transformed into nineteen bioclimatic variables utilizing the “biovars” function within the “dismo” package of R software (version 4.2.2) [31,38]. Following the extraction of environmental variables at the occurrence coordinates of *L. boeticus*, Spearman’s correlation coefficient was computed using IBM SPSS Statistics (version 21, IBM Statistics, Armonk, NY, USA) [39]. For variable selection, those with correlation coefficients exceeding ±0.7 were removed in favor of key variables that contributed significantly to the default MaxEnt model, which incorporated 20 environmental variables [40]. Subsequently, bioclimatic variables associated with high temperature, humidity, and precipitation were taken into account, as these factors influence *L. boeticus* populations [1,41,42]. Nine environmental variables (Bio2, 6, 8, 13, 14, 15, 18, and 19, and elevation) were selected for *L. boeticus*. The relative importance of these variables was assessed using the percentage contribution (for MaxEnt) and the mean decrease in Gini (for RF) [43]. To model climate change scenarios, the Shared Socioeconomic Pathway (SSP) 5-8.5 for the period 2081–2100 from the MIROC-6 sourced from WorldClim at a 10 arc minute resolution [35,44]. The SSP5-8.5 scenario was employed to construct the models under the assumption of an extreme simulation, which forecasts a global mean temperature increase of up to 5.1 °C, alongside variable precipitation patterns [45]. The timeframe of 2081–2100 was selected because it provides an appropriate duration for observing changes in future pest distribution, being neither excessively short nor overly extended.

### 2.3. Optimal Model Operating Conditions

An optimal model operation is required to develop a high-performance model along with the selection of variables [46]. Therefore, we determined the ideal operational parameters for both MaxEnt and RF. For MaxEnt, we used the ENMeval package in R to identify the optimal regularization multiplier (RM) and feature combination (FC) based on environmental data [47]. The optimal RM and FC for *L. boeticus* were determined to be 2.5 and the linear-quadratic-hinge-product (LQHP) feature, respectively, which showed the smallest ∆ AICc (delta corrected Akaike information criterion). Following the identification of the optimal parameters for the MaxEnt operation, we implemented models for *L. boeticus* using 10-fold cross-validation with 10,000 background points [21].

For the RF algorithm, pseudo-absence data were generated at a 1:1 ratio with presence data using the mopa package [48]. The data were partitioned into a 70% training set and a 30% testing set. The number of variables randomly sampled at each split (mtry) was set to 3 (the square root of the number of environmental variables), and the number of trees (ntree) was set to 500, a value at which the out-of-bag (OOB) error rate stabilized [25,49].

### 2.4. Model Performance Test

Model performance was assessed using three standard metrics: accuracy, the area under the receiver operating characteristic curve (AUC), and the true skill statistic (TSS). Accuracy represents the proportion of correct predictions. AUC values were interpreted using a standard scale: 0.5–0.6 (fail), 0.6–0.7 (poor), 0.7–0.8 (fair), 0.8–0.9 (good), and >0.9 (excellent) [50]. TSS is a prevalence-independent metric of accuracy; its values were interpreted as follows: 0–0.2 (no better than random), 0.2–0.4 (poor), 0.4–0.6 (fair), 0.6–0.8 (good), and >0.8 (excellent) [51,52]. The TSS was calculated using the maximum training sensitivity plus specificity logistic threshold, which provides a robust and prevalence-independent cutoff [53].

## 3. Results

### 3.1. Results of Model Performance Test for L. boeticus

The developed models demonstrated fair predictive performance, though their metrics and spatial predictions varied (Table 1). The RF model consistently achieved higher performance values (AUC, accuracy, and TSS > 0.8) compared to the MaxEnt model. This difference may be attributed to RF’s more constrained predictions around known occurrence points, resulting in higher specificity on the test data. In contrast, MaxEnt predicted a broader potential distribution, which, despite having a similar sensitivity to RF, led to lower overall performance scores.

### 3.2. Occurrence, Variables, and Potential Distribution of L. boeticus by Single Model Algorithm

The total *L. boeticus* dataset of 3669 points for model development were used with nine environmental variables (Bio2, 6, 8, 13, 14, 15, 18, and 19, and elevation). As a result of percent contribution, the minimum temperature in the coldest month was the most significant factor across the models (Table 2).

**Figure 2:**
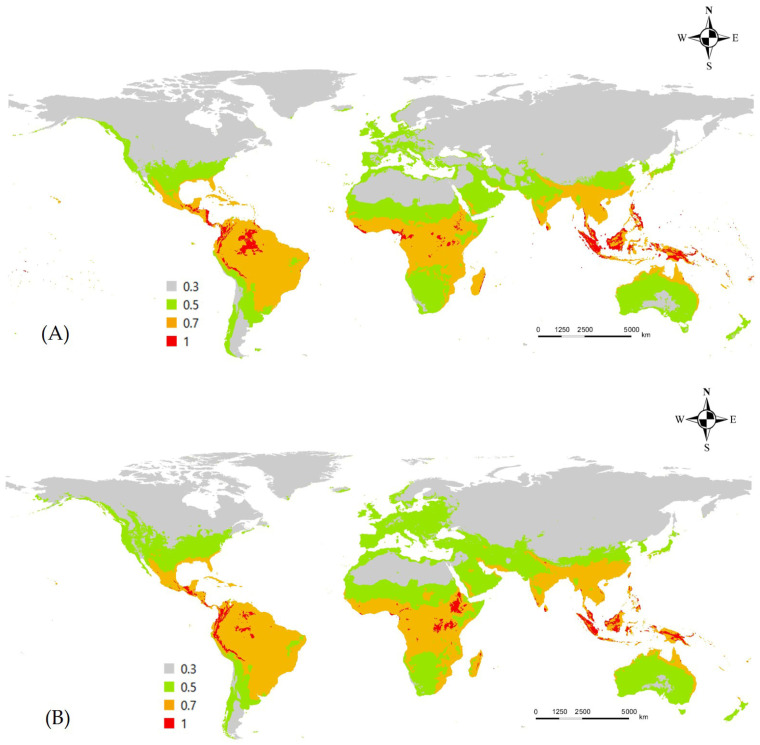
Potential distribution of *L. boeticus* under current/future climate in MaxEnt model. (**A**) Current climate, (**B**) future climate (2081–2100).

The MaxEnt model predicted high habitat suitability for *L. boeticus* across most tropical and subtropical regions, as well as some temperate zones. The primary contributing environmental factors were the minimum temperature of the coldest month (bio6), precipitation of the wettest month (bio13), and precipitation of the warmest quarter (bio16) identified as the key contributing environmental factors. The optimal habitat latitude range was identified as approximately −15 to 15 degrees, encompassing Central and South America where its occurrence has not been previously reported. Under the SSP5-8.5 climate scenario, the potential distribution of the pest is projected to expand northward in the Northern Hemisphere (Figure 2).

**Figure 3:**
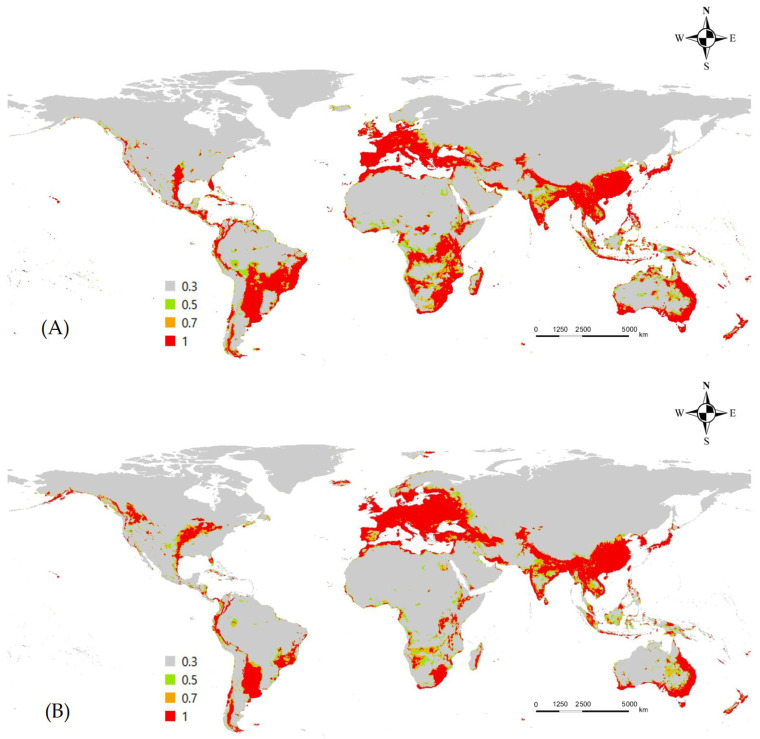
Potential distribution of *L. boeticus* under current/future climate in random forest model. (**A**) Current climate, (**B**) future climate (2081–2100).

The RF model also predicted high suitability in tropical, subtropical, and temperate regions. However, the key contributing factors were slightly different: the minimum temperature of the coldest month (bio6), precipitation of the coldest quarter (bio19), and the mean diurnal range (bio2). Compared to MaxEnt, the predicted optimal habitat range was much narrower. Notably, some equatorial regions (e.g., Borneo) and the Australian Outback showed low suitability, creating clearer boundaries between suitable and unsuitable areas. Conversely, RF predicted notably higher suitability across Europe and an extensive suitable region in the southwestern United States. Under the SSP5-8.5 scenario, the model projected a similar northward expansion in the Northern Hemisphere, but also a decline in habitat suitability in parts of South America and Southern Africa (Figure 3).

### 3.3. Ensemble Prediction of L. boeticus Potential Distribution with Consideration of Host Production and Global Pulse-Crop Production Area with Potential Risk Area Assessment

**Figure 4:**
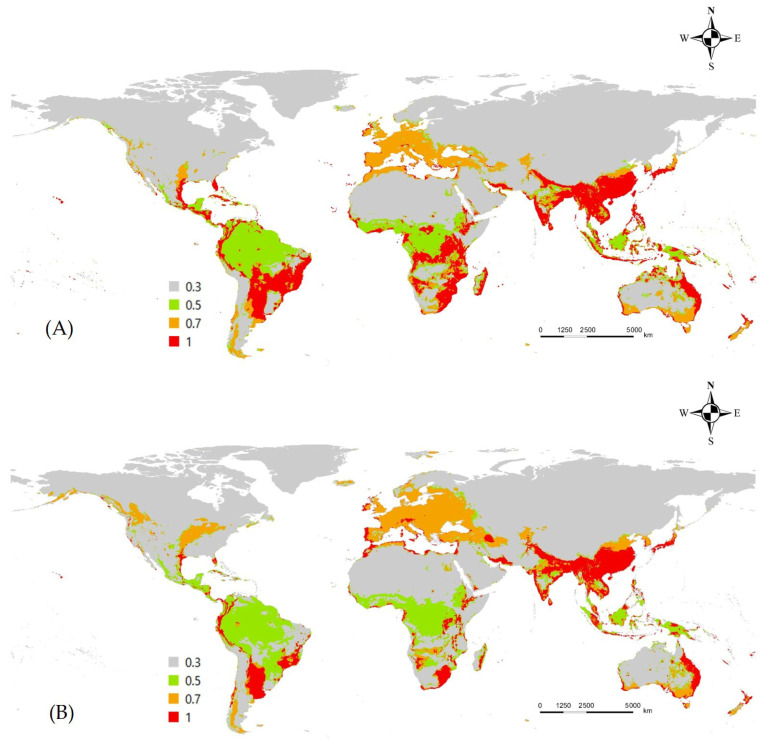
Average based ensemble model of potential distribution of *L. boeticus* under current/future climate. (**A**) Current climate, (**B**) future climate (2081–2100).

The ensemble model, which averages the predictions, showed a reduction in the area classified as “very highly suitable” (suitability > 0.7) but an increase in “high” (0.5–0.7) and “medium” (0.3–0.5) suitability areas. This smoothing effect was particularly evident in Europe, reflecting the broader predictions of the MaxEnt model. According to the ensemble model, the primary risk areas for this pest include India, China, Southeast Asia, parts of Australia and New Zealand, most of Europe, and parts of Africa and the Americas. While most of these areas are predicted to remain suitable under climate change, regions in South America and Africa may see a decline. Conversely, the Baltic countries, Ukraine, and several U.S. states (Kansas, Missouri, Iowa, Illinois, and Michigan) are projected to become significantly more suitable in the future (Figure 4).

**Figure 5:**
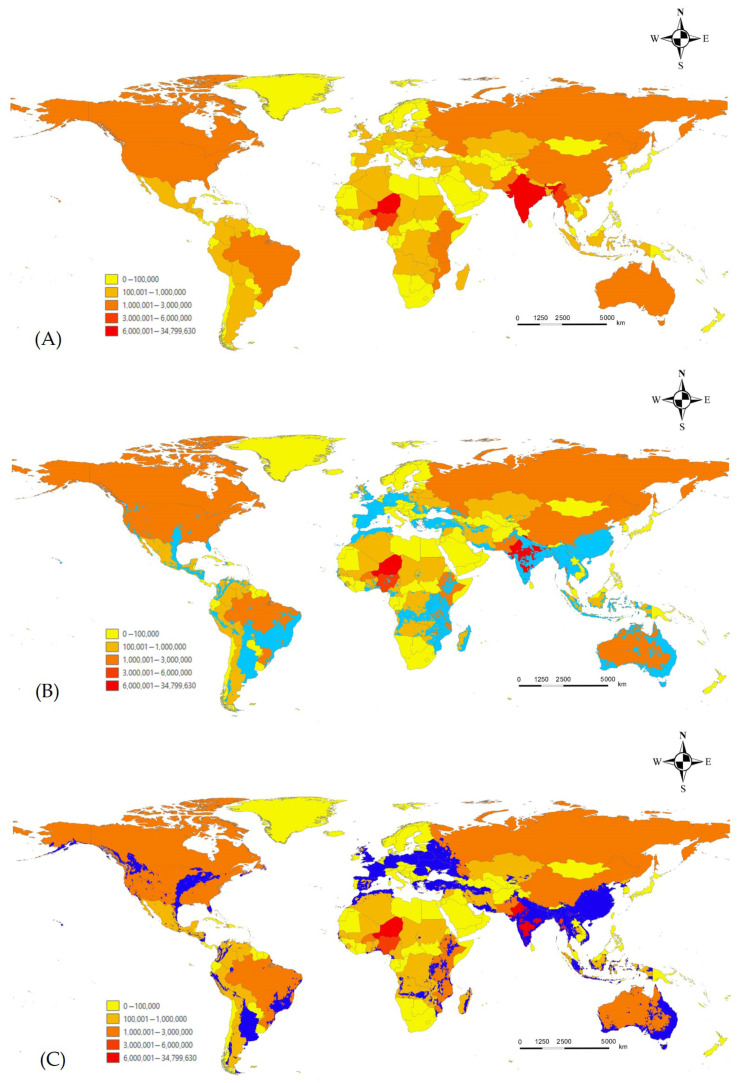
Global pulse-crop production area and potential risk area assessment. (**A**) Global pulse crop area harvested (ha) in 2023, (**B**) current suitability areas of *L. boeticus* with countries have pulse crop harvested areas exceeding 100,000 hectares, and (**C**) future suitability areas of *L. boeticus* with countries have pulse crop harvested areas exceeding 100,000 hectares.

To evaluate agricultural risk, we overlaid the predicted potential distribution (ensemble model; suitability > 0.5) with data on pulse-crop cultivation. Countries with over 1 million hectares of pulse cultivation that also fall within areas of high habitat suitability for *L. boeticus* include India, Pakistan, China, Myanmar, Australia, the United States, Brazil, and several African nations [54]. Under the future climate scenario, these risk areas are projected to expand northward in North America and Eurasia. In contrast, a significant reduction in risk is projected for southeastern South America and parts of Africa (Figure 5).

## 4. Discussion

In this study, we developed and compared MaxEnt and RF models for *L. boeticus* and projected their potential distributions according to climate change. The model performance metrics of AUC and TSS showed fair effectiveness in predicting the target species, meeting the standard criteria for the metrics. This suggests that the developed models adequately explain the possibility of the occurrence of the species. However, the RF model showed much better performance in terms of AUC, accuracy, and TSS than the MaxEnt model. As the occurrence coordinates of *L. boeticus* were relatively abundant in Europe, the MaxEnt model was influenced more by data bias, even with spatial filtering. In contrast, the performance index of the RF model, which effectively utilized pseudo-absence data, demonstrated a comparatively superior performance level. Regardless of the evaluation index employed, several island areas (Ascension, Saipan, Chuuk, Marshall, and Norfolk) that exhibit high habitat suitability despite the absence of coordinates used in the model have been confirmed in the literature, allowing for the verification of model performance in areas without coordinates [55,56,57,58].

Among the environmental variables, the minimum temperature in the coldest month was the most influential regardless of the model. This finding aligns with the ecological profile of *L. boeticus*, a species primarily found in tropical and subtropical zones and have limitations in northern and southern areas whose range is constrained by its thermal developmental thresholds [41,42]. It was assumed that variables other than the minimum temperature of the coldest month, which had the greatest influence, showed different results owing to differences in the model algorithms. The precipitation of the wettest month or warmest/coldest quarter also considerably influenced the models, which is consistent with previous studies indicating substantial impacts of precipitation and humidity on *L. boeticus* species [42,59,60,61]. Additionally, the mean diurnal range considerably influenced the RF model, which is consistent with the results of other lepidoptran species distribution studies [62,63,64]. The survival of a butterfly is substantially impacted by body temperature requiring them to regulate their body temperature within an appropriate range [63,65]. As small ectothermic animals, butterflies are highly reliant on their environment for thermoregulation, making them particularly sensitive to fluctuations in temperature [63,66].

Spatially, both models identified high suitability across a broad spectrum of tropical, subtropical, and temperate regions, largely aligning with the coordinates of *L. boeticus*, CABI, and the distribution literature [1,26,27]. Suitability was highest in India, where the pest causes the most reported damage. However, it also exhibited high suitability in some regions of China and Southeast Asia. The MaxEnt model tended to underestimate suitability in Europe and overestimate it in Australia. In contrast, the RF model appears to underestimate the suitability in the equatorial regions. The limitations of each model were addressed through an average-based ensemble approach, resulting in a model that reflected a more realistic suitability pattern.

Given its strong migratory ability, this species is expected to encounter minimal habitat restrictions, aside from climatic factors, owing to its diverse range of host plants. Notably, it predominantly feeds on legumes as its primary food source among the various available host plants. India is one of the world’s top pulse-producing countries, along with China, Myanmar, the United States, and Brazil, which exhibit high habitat suitability for *L. boeticus* [54]. Within the Old Continent, where *L. boeticus* is distributed, China, Myanmar, and India are countries with high-pulse cultivation. However, instances of damage have not been reported as much as in India [67,68]. In Myanmar and China, which have favorable climatic conditions, the population of *L. boeticus* is believed to be regulated by interspecific competition, natural enemies, and other biological factors to a different degree than in India. If this species were to invade countries with high-pulse cultivation areas, such as the United States, Brazil, Argentina, and Mexico, where it is not currently present, the potential for significant damage is high. Particularly in Central America, including the south-central United States (Florida, Texas, Oklahoma), eastern Mexico, and South America, where the habitat is highly suitable, the risk of damage is considerable in parts of Venezuela, Colombia, Ecuador, Peru, Bolivia, Paraguay, eastern Argentina, and southeastern Brazil. In addition, it is important to be cautious when considering the expansion of suitable habitats for *L. boeticus* to the northeastern United States, northeastern South America, and northeastern Europe, as these areas are likely to be at risk due to climate change. This species, with a serious impact on pulse-crop cultivation, is expected to have more outbreaks because of climate change. Therefore, quarantine is crucial in countries where this species is not present, while countries where it has already established should prepare for management using strategies including the application of pesticides and resistant varieties [9,12,69].

In the present study, we assessed the potential distribution of *L. boeticus* and identified regions vulnerable to damage in relation to climatic variables. Our analysis indicates specific areas with favorable climatic conditions for this species, highlighting the need for cautious monitoring to prevent invasion. This study provides fundamental data for creating effective management strategies.

## Figures and Tables

**Table 1:** Measures of the models for *L. boeticus*.

Measure	MaxEnt	Random Forest
Test AUC	0.6694	0.9015
Accuracy	0.6545	0.9012
TSS	0.5155	0.8027

**Table 2:** List of selected environmental variables for *L. boeticus*.

Variable Code ^a^	Description	Percent Contribution (MaxEnt)	Mean Decrease Gini (Random Forest)
Bio2	Mean diurnal range ^a,b^	0.3	264.94
Bio6	Minimum temperature of the coldest month	59	853.61
Bio8	Mean temperature of the wettest quarter	2	210.48
Bio13	Precipitation of wettest month	16	251.73
Bio14	Precipitation of driest month	0.2	149.53
Bio15	Precipitation seasonality (Coefficient of variation)	2.8	132.13
Bio18	Precipitation of warmest quarter	15.1	182.77
Bio19	Precipitation of the coldest quarter	1.6	375.96
Elevation	Altitude data	2.9	129.76

^a^ Variable codes were obtained from WorldClim (www.worldclim.org). ^b^ Mean diurnal range = mean of monthly (maximum temperature–minimum temperature).

## Data Availability

Data is contained within the article.
